# Supplementary figures and images for: Hardware implementation of FPGA-based spiking attention neural network accelerator
Source: PeerJ Comput Sci. 2025 Aug 5;11:e3077. doi: 10.7717/peerj-cs.3077 (PMC12453718; doi:10.7717/peerj-cs.3077)

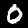

Supplement: Supplemental Information 1 — The files are based on SNN.zip which mainly holds the code for training on the software side and HLS.rar which mainly holds the implementation code on the FPGA side. The code for the article was built based on the Snntorch library. The required environment is python > 3.9. PyTorch should be installed to use snnTorch. Ensure the correct version of torch is installed for your system to enable CUDA compatibility. The following packages are automatically installed if using the pip command: • numpy • pandas The following packages are required for using export_nir and import_nir: • nir • nirtorch The following packages are required for using spikeplot: • matplotlib Our inference code is placed in SNN_code\snntorch_demo\inference. weight, weight2, and weight3 are our weight export files, respectively. [file peerj-cs-11-3077-s001.zip › img/0img.bmp]

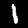

Supplement: Supplemental Information 1 — The files are based on SNN.zip which mainly holds the code for training on the software side and HLS.rar which mainly holds the implementation code on the FPGA side. The code for the article was built based on the Snntorch library. The required environment is python > 3.9. PyTorch should be installed to use snnTorch. Ensure the correct version of torch is installed for your system to enable CUDA compatibility. The following packages are automatically installed if using the pip command: • numpy • pandas The following packages are required for using export_nir and import_nir: • nir • nirtorch The following packages are required for using spikeplot: • matplotlib Our inference code is placed in SNN_code\snntorch_demo\inference. weight, weight2, and weight3 are our weight export files, respectively. [file peerj-cs-11-3077-s001.zip › img/1img.bmp]

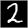

Supplement: Supplemental Information 1 — The files are based on SNN.zip which mainly holds the code for training on the software side and HLS.rar which mainly holds the implementation code on the FPGA side. The code for the article was built based on the Snntorch library. The required environment is python > 3.9. PyTorch should be installed to use snnTorch. Ensure the correct version of torch is installed for your system to enable CUDA compatibility. The following packages are automatically installed if using the pip command: • numpy • pandas The following packages are required for using export_nir and import_nir: • nir • nirtorch The following packages are required for using spikeplot: • matplotlib Our inference code is placed in SNN_code\snntorch_demo\inference. weight, weight2, and weight3 are our weight export files, respectively. [file peerj-cs-11-3077-s001.zip › img/2img.bmp]

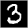

Supplement: Supplemental Information 1 — The files are based on SNN.zip which mainly holds the code for training on the software side and HLS.rar which mainly holds the implementation code on the FPGA side. The code for the article was built based on the Snntorch library. The required environment is python > 3.9. PyTorch should be installed to use snnTorch. Ensure the correct version of torch is installed for your system to enable CUDA compatibility. The following packages are automatically installed if using the pip command: • numpy • pandas The following packages are required for using export_nir and import_nir: • nir • nirtorch The following packages are required for using spikeplot: • matplotlib Our inference code is placed in SNN_code\snntorch_demo\inference. weight, weight2, and weight3 are our weight export files, respectively. [file peerj-cs-11-3077-s001.zip › img/3img.bmp]

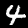

Supplement: Supplemental Information 1 — The files are based on SNN.zip which mainly holds the code for training on the software side and HLS.rar which mainly holds the implementation code on the FPGA side. The code for the article was built based on the Snntorch library. The required environment is python > 3.9. PyTorch should be installed to use snnTorch. Ensure the correct version of torch is installed for your system to enable CUDA compatibility. The following packages are automatically installed if using the pip command: • numpy • pandas The following packages are required for using export_nir and import_nir: • nir • nirtorch The following packages are required for using spikeplot: • matplotlib Our inference code is placed in SNN_code\snntorch_demo\inference. weight, weight2, and weight3 are our weight export files, respectively. [file peerj-cs-11-3077-s001.zip › img/4img.bmp]

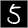

Supplement: Supplemental Information 1 — The files are based on SNN.zip which mainly holds the code for training on the software side and HLS.rar which mainly holds the implementation code on the FPGA side. The code for the article was built based on the Snntorch library. The required environment is python > 3.9. PyTorch should be installed to use snnTorch. Ensure the correct version of torch is installed for your system to enable CUDA compatibility. The following packages are automatically installed if using the pip command: • numpy • pandas The following packages are required for using export_nir and import_nir: • nir • nirtorch The following packages are required for using spikeplot: • matplotlib Our inference code is placed in SNN_code\snntorch_demo\inference. weight, weight2, and weight3 are our weight export files, respectively. [file peerj-cs-11-3077-s001.zip › img/5img.bmp]

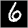

Supplement: Supplemental Information 1 — The files are based on SNN.zip which mainly holds the code for training on the software side and HLS.rar which mainly holds the implementation code on the FPGA side. The code for the article was built based on the Snntorch library. The required environment is python > 3.9. PyTorch should be installed to use snnTorch. Ensure the correct version of torch is installed for your system to enable CUDA compatibility. The following packages are automatically installed if using the pip command: • numpy • pandas The following packages are required for using export_nir and import_nir: • nir • nirtorch The following packages are required for using spikeplot: • matplotlib Our inference code is placed in SNN_code\snntorch_demo\inference. weight, weight2, and weight3 are our weight export files, respectively. [file peerj-cs-11-3077-s001.zip › img/6img.bmp]

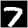

Supplement: Supplemental Information 1 — The files are based on SNN.zip which mainly holds the code for training on the software side and HLS.rar which mainly holds the implementation code on the FPGA side. The code for the article was built based on the Snntorch library. The required environment is python > 3.9. PyTorch should be installed to use snnTorch. Ensure the correct version of torch is installed for your system to enable CUDA compatibility. The following packages are automatically installed if using the pip command: • numpy • pandas The following packages are required for using export_nir and import_nir: • nir • nirtorch The following packages are required for using spikeplot: • matplotlib Our inference code is placed in SNN_code\snntorch_demo\inference. weight, weight2, and weight3 are our weight export files, respectively. [file peerj-cs-11-3077-s001.zip › img/7img.bmp]

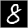

Supplement: Supplemental Information 1 — The files are based on SNN.zip which mainly holds the code for training on the software side and HLS.rar which mainly holds the implementation code on the FPGA side. The code for the article was built based on the Snntorch library. The required environment is python > 3.9. PyTorch should be installed to use snnTorch. Ensure the correct version of torch is installed for your system to enable CUDA compatibility. The following packages are automatically installed if using the pip command: • numpy • pandas The following packages are required for using export_nir and import_nir: • nir • nirtorch The following packages are required for using spikeplot: • matplotlib Our inference code is placed in SNN_code\snntorch_demo\inference. weight, weight2, and weight3 are our weight export files, respectively. [file peerj-cs-11-3077-s001.zip › img/8img.bmp]

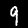

Supplement: Supplemental Information 1 — The files are based on SNN.zip which mainly holds the code for training on the software side and HLS.rar which mainly holds the implementation code on the FPGA side. The code for the article was built based on the Snntorch library. The required environment is python > 3.9. PyTorch should be installed to use snnTorch. Ensure the correct version of torch is installed for your system to enable CUDA compatibility. The following packages are automatically installed if using the pip command: • numpy • pandas The following packages are required for using export_nir and import_nir: • nir • nirtorch The following packages are required for using spikeplot: • matplotlib Our inference code is placed in SNN_code\snntorch_demo\inference. weight, weight2, and weight3 are our weight export files, respectively. [file peerj-cs-11-3077-s001.zip › img/9img.bmp]
